# Validity over time of self-reported anthropometric variables during follow-up of a large cohort of UK women

**DOI:** 10.1186/s12874-015-0075-1

**Published:** 2015-10-08

**Authors:** F. Lucy Wright, Jane Green, Gillian Reeves, Valerie Beral, Benjamin J. Cairns, on behalf of the Million Women Study collaborators

**Affiliations:** Cancer Epidemiology Unit, Nuffield Department of Population Health, University of Oxford, Richard Doll Building, Old Road Campus, Headington, Oxford, OX3 7LF UK

**Keywords:** Anthropometry, Body size, Longitudinal study, Self-report, Validation

## Abstract

**Background:**

In prospective epidemiological studies, anthropometry is often self-reported and may be subject to reporting errors. Self-reported anthropometric data are reasonably accurate when compared with measurements made at the same time, but reporting errors and changes over time in anthropometric characteristics could potentially generate time-dependent biases in disease-exposure associations.

**Methods:**

In a sample of about 4000 middle-aged UK women from a large prospective cohort study, we compared repeated self-reports of weight, height, derived body mass index, and waist and hip circumferences, obtained between 1999 and 2008, with clinical measurements taken in 2008. For self-reported and measured values of each variable, mean differences, correlation coefficients, and regression dilution ratios (which measure relative bias in estimates of linear association) were compared over time.

**Results:**

For most variables, the differences between self-reported and measured values were small. On average, reported values tended to be lower than measured values (i.e. under-reported) for all variables except height; under-reporting was greatest for waist circumference. As expected, the greater the elapsed time between self-report and measurement, the larger the mean differences between them (each *P* < 0.001 for trend), and the weaker their correlations (each *P* < 0.004 for trend). Regression dilution ratios were in general close to 1.0 and did not vary greatly over time.

**Conclusion:**

Reporting errors in anthropometric variables may result in small biases to estimates of associations with disease outcomes. Weaker correlations between self-reported and measured values would result in some loss of study power over time. Overall, however, our results provide new evidence that self-reported anthropometric variables remain suitable for use in analyses of associations with disease outcomes in cohort studies over at least a decade of follow-up.

**Electronic supplementary material:**

The online version of this article (doi:10.1186/s12874-015-0075-1) contains supplementary material, which is available to authorized users.

## Background

In prospective epidemiological studies, exposure information is often self-reported and may be subject to reporting errors, and thus may potentially bias estimates of association with disease outcomes. Furthermore, exposures may change over time, which could further compound the role of measurement error after long-term follow-up. Previous studies have found that self-reported anthropometric variables are reasonably accurate when compared with measurements made at the same time, and are generally adequate for use in large-scale epidemiological studies [1–10]. However, such comparisons do not allow for changes over time, which could potentially generate time-dependent biases in estimates of disease-exposure associations. Validity over time of repeated anthropometric variables has been examined for predominantly measured values [11, 12], but not separately and specifically for self-reported values.

The aim of this study was to assess the accuracy over time of self-reported anthropometric characteristics (weight, height, derived body mass index (BMI), waist and hip circumference) during follow-up of the Million Women Study, a large UK cohort of women in middle age. We compared self-reported data at four different times over 9 years with measured values from a single examination conducted an average of 9 years after recruitment.

## Methods

### Setting: Million Women Study

Between 1996 and 2001, 1.3 million women aged 50–64 years (56 years, on average) were recruited to the Million Women Study through UK National Health Service (NHS) Breast Screening Centres in England and Scotland [13]. At recruitment (mean year, 1998), women completed a self-administered health and lifestyle questionnaire which included questions on their weight and height. All women were sent resurvey questionnaires at approximately 3–5 yearly intervals; these included questions about their current weight as well as their waist and hip circumferences. Study questionnaires and further details of the data and access policies can be viewed on the website (www.millionwomenstudy.org).

At recruitment, all study participants gave written consent to participate in medical research and to be contacted in the future. Ethical approval for the study was obtained from the Oxford and Anglia Multi-Centre Research Ethics Committee. All study participants have a unique NHS number. Using this and other identifying details, they are followed up for deaths, emigration, cancer registrations, and changes in name, address and registered general practitioner, through electronic linkage with the NHS Central Registers.

### Study sample and data collection

The present analysis included a sample of women from the Million Women Study with self-reported anthropometric data over 9 years and a single clinical measurement at the end of this period (Table 1). From the main study, self-reports were available from the first (recruitment) questionnaire (mean year, 1999), the second questionnaire (mean year, 2002) and the third questionnaire (mean year, 2006). (All questionnaires and the clinical measurement will henceforth be referred to by the mean year in which they were completed by participants in this validation study.) In 2008, as part of the collection of blood samples in randomly selected participants in the Million Women Study, an additional questionnaire was sent to 14,762 women who had responded to the 2006 study questionnaire, and had not reported breast cancer or vascular disease. All were asked about their current weight and height, and women resident in England only were also asked about their current waist and hip circumferences. Amongst all selected women, 5975 indicated that they were willing to attend a general practice appointment for clinical investigations. In total 3999 (67 % of 5975) women had their anthropometric characteristics measured at their general practice, returned a completed form with the measurements recorded, and had usable reported values. These women form the study population for the present analyses. Additional details of inclusion criteria and data collection methods are given in the Methods supplement in the Additional file 1.

**Table 1:** Timeline of collection of anthropometric variables

	Year (mean)	Anthropometric variables collected
1st (recruitment) questionnaire^a^	1999	Self-reported weight and height
2nd questionnaire^a^	2002	Self-reported weight, waist and hips
3rd questionnaire^a^	2006	Self-reported weight, waist and hips
Additional questionnaire	2008	Self-reported weight, height, waist^b^ and hips^b^
Examination	2008	Measured weight, height, waist^b^ and hips^b^

^a)^See www.millionwomenstudy.org/questionnaires

^b)^Women resident in England only

Self-reported values for anthropometric variables from the recruitment questionnaire in 1999 and two subsequent study questionnaires in 2002 and 2006 were obtained from the Million Women Study database. All included women had, by definition, responded to the recruitment questionnaire in 1999, the third study questionnaire in 2006 and the additional questionnaire in 2008, and the great majority (92 %, 3691) had also responded to the second study questionnaire in 2002. For weight and height, data were virtually complete for both reported (97 and 99 %, respectively) and measured (≥99 % for each) values of these characteristics (Web Table 1). A somewhat smaller proportion of women reported their waist or hip circumferences (75 %, for each), while measured values were, again, virtually complete (≥99 %). Body mass index (BMI) was derived for each questionnaire and the clinical measurement as the weight (kg) divided by the square of the height (m). Since women were not asked to report their height on the 2002 and 2006 questionnaires, BMIs for those questionnaires were calculated using height values reported on the 1999 questionnaire. As for height and weight, the BMI values were virtually complete from both questionnaires and the measurements (97 % and > 99 %, respectively).

### Data analysis

Reported values from the 1999, 2002, 2006 and 2008 questionnaires were compared in several ways with the measured values from 2008. For each variable, the difference between mean reported and mean measured values was assessed using a paired *t*-test; 95 % confidence intervals (CI) were calculated for estimates appearing in the text. Pearson correlation coefficients were computed to investigate the strength of associations between self-reports and measured values. Pearson correlation coefficients also estimate the loss of power due to reporting errors: the square of the correlation coefficient is an estimate of the effective sample size relative to the apparent sample size for power calculations [14, 15]. Regression dilution ratios were calculated by linear regression of the measured values against reported values from each questionnaire [16]. Regression dilution ratios estimate the relative attenuation of regression coefficients (e.g., relative biases in log relative risks) due to systematic and random reporting errors or changes in characteristics over time. Thus a regression dilution ratio provides an overall summary of the expected bias in epidemiological studies as a consequence of reporting errors as well as any changes over time in the characteristic of interest (including changes due to population-level trends and the natural history of advancing age). P-values for trends over time in either mean differences or regression dilution ratios were calculated by generalised least squares. Covariance matrices were estimated for the values for each questionnaire from 10,000 replicates of the bootstrap sampling procedure.

Continuous anthropometric variables are commonly categorised in epidemiological analyses, so we also investigated how measured values varied over time within categories of self-reported values. For each self-reported anthropometric variable on each questionnaire, five categories were created using pre-specified cut points [10]. We calculated means of the measured values of each anthropometric variable (taken at the single examination in 2008, on average) within each of the five categories of the corresponding self-reported variables (taken from the 1999, 2002, 2006 and 2008 questionnaires). These mean measured values were plotted for each variable, to assess the stability of reported values over the 9-year follow-up period. Analyses were adjusted for attained age at the examination and for recruitment region. All analyses were performed using Stata version 12.0.

## Results

There were 3999 women from the Million Women Study cohort included in this study. Table 2 summarises the anthropometric data from four study questionnaires administered over an average of 9 years (1999 to 2008), and anthropometry measured at an examination in 2008, 9 years on average after recruitment. At the time of the examination, the mean age of the women was 66 years (SD 4.7). Overall, there were slight increases over time in mean reported values for all anthropometric variables, with the exception of height, which showed a slight decline. Generally, means and standard deviations of self-reported anthropometric variables on each questionnaire (Table 2) were very similar to corresponding values reported by the entire Million Women Study cohort (Web Table 2), suggesting that women in this validation study are representative of the cohort as a whole.

**Table 2:** Self-reported and measured anthropometric characteristics of 3999 women in the study sample

	Mean year of report				Mean year of measurement
	1999	2002^a^	2006	2008	2008
Age at time of report or measurement, mean (SD)	56.1 (4.6)	60.1 (4.7)	64.1 (4.6)	65.7 (4.7)	66.0 (4.7)
Anthropometry:					
Weight, kg, mean (SD)	67.1 (11.2)	67.6 (11.5)	68.4 (11.9)	68.4 (11.9)	69.8 (12.6)
Height, cm, mean (SD)^b^	162.7 (6.5)	NA	NA	162.2 (6.3)	161.5 (6.3)
BMI, kg/m^2^, mean (SD)^b^	25.4 (4.1)	25.5 (4.2)	25.8 (4.4)	26.0 (4.4)	26.8 (4.7)
Waist, cm, mean (SD)	NA	76.8 (9.3)	79.3 (10.0)	82.2 (10.3)	88.8 (12.6)
Hips, cm, mean (SD)	NA	100.3 (7.5)	100.6 (7.8)	101.6 (8.2)	104.7 (10.3)

Women with missing values for a variable were excluded when calculating the means

*Abbreviations*: *BMI* body mass index, *NA* not asked, *SD* standard deviation

^a)^3691 of 3999 women responded to this questionnaire

^b)^BMI values for 2002 and 2006 were calculated using height reported in 1999

Overall, self-reported anthropometry from all four questionnaires compared well with measured values from the examination (Table 3). For all variables except height, the mean of the first report (in either 1999 or 2002) was lower than the mean of the measured value in 2008 (*P* < 0.001). This difference was greatest for waist circumference between first report in 2002 and measurement in 2008 (mean difference 10.3 cm, 95 % CI: 9.9, 10.7). For height the mean of the first reported value (in 1999) was greater than the measured value (by 1.2 cm on average, 95 % CI: 1.1 to 3.1). From the earliest to the latest self-reports, the mean difference between the reported values and measurement tended to attenuate towards 0 for all variables; i.e., differences were smaller, the shorter the elapsed time between self-report and the measurement (*P* < 0.001 for trend for all variables).

**Table 3:** Comparisons between measured values in 2008 and earlier self-reported values

Characteristic Mean year of report^a^	N	Mean difference (se)^b^ Reported – measured	Pearson correlation	Regression dilution ratio (95 % CI)^c^
Weight (kg)				
1999	3893	−2.6 (0.10)	0.88	1.00 (0.98–1.01)
2002	3530	−1.8 (0.09)	0.91	0.99 (0.98–1.00)
2006	3870	−1.2 (0.07)	0.94	0.99 (0.98–1.00)
2008	3921	−1.1 (0.05)	0.97	1.02 (1.01–1.02)
P for trend		<0.001	<0.001	0.07
Height (cm)^d^				
1999	3951	1.2 (0.05)	0.88	0.85 (0.84–0.87)
2008	3943	0.8 (0.04)	0.91	0.89 (0.88–0.90)
P for trend		<0.001	0.003	0.02
BMI (kg/m ^2^ ) ^d^				
1999	3863	−1.4 (0.04)	0.85	0.98 (0.96–1.00)
2002	3496	−1.1 (0.04)	0.88	0.96 (0.95–0.98)
2006	3834	−0.9 (0.03)	0.91	0.97 (0.96–0.99)
2008	3887	−0.7 (0.02)	0.95	1.01 (1.00–1.02)
P for trend		<0.001	<0.001	0.30
Waist (cm)^e^				
2002	1771	−10.3 (0.21)	0.66	0.84 (0.80–0.88)
2006	1717	−7.2 (0.21)	0.68	0.78 (0.74–0.82)
2008	2153	−5.2 (0.18)	0.71	0.80 (0.77–0.83)
P for trend		<0.001	<0.001	0.08
Hips (cm)^e^				
2002	1774	−2.8 (0.15)	0.75	0.95 (0.91–0.99)
2006	1724	−2.3 (0.15)	0.76	0.90 (0.87–0.94)
2008	2165	−2.0 (0.12)	0.82	0.95 (0.92–0.97)
P for trend		<0.001	<0.001	0.70

*Abbreviations*: *BMI* body mass index, *CI* confidence interval, *RDR* regression dilution ratio, *se* standard error

^a)^Mean elapsed time between reporting and measurement in 2008: 9 years (reported in 1999), 6 years (reported in 2002), 2 years (reported in 2006) and 4 months (reported in 2008)

^b)^Difference = reported minus measured in 2008, negative values represent under-reporting and positive values are over-reporting

^c)^Adjusted for age at measurement and recruitment region

^d)^Women were not asked for their height in 2002 or 2006. BMI values were calculated using height reported in 1999

^e)^Women were not asked for waist and hip circumference in 1999

Self-reported anthropometric variables had moderately strong to very strong correlations with the measurements (Table 3). For weight, height and BMI, Pearson correlations between self-reports and measured values ranged between 0.85 and 0.95 during the 9-year study period. For waist and hip circumferences, Pearson correlations were somewhat weaker, ranging between 0.66 and 0.82. Comparing correlation coefficients with measured values from first report to the 2008 questionnaire, there were higher correlations at shorter elapsed times between the questionnaire and the examination (weight, derived body mass index, waist and hip circumferences: *P* < 0.001 for trend; height: *P* = 0.003 for trend).

Calculated regression dilution ratios indicated that reporting errors are likely to generate little bias in estimates of associations with disease outcomes (Table 3). The regression dilution ratios were 1.02 for weight, 0.85 for height, 0.98 for BMI, 0.84 for waist circumference and 0.95 for hip circumference at the time the variables were first reported. Regression dilution ratios remained relatively constant over time between self-report and the measurement, suggesting that changes over time in these anthropometric characteristics are likely to generate little time-dependent regression dilution bias (Table 3).

**Fig. 1:**
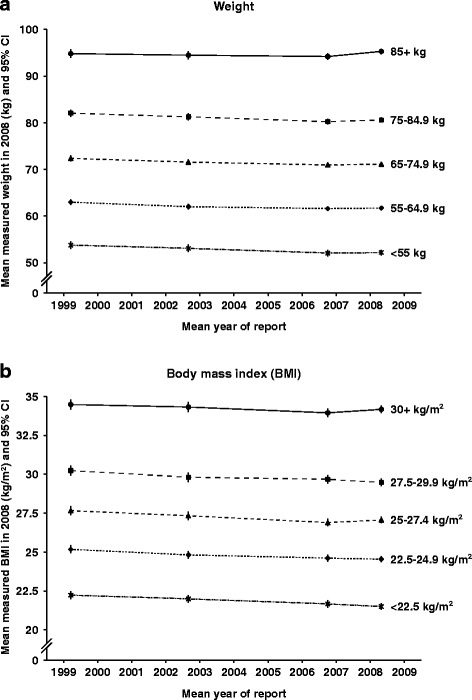
Mean measured values in 2008 by categories of self-reported values on four different occasions (1999, 2002, 2006, 2008) (**a**) weight, (**b**) body mass index

**Fig. 2:**
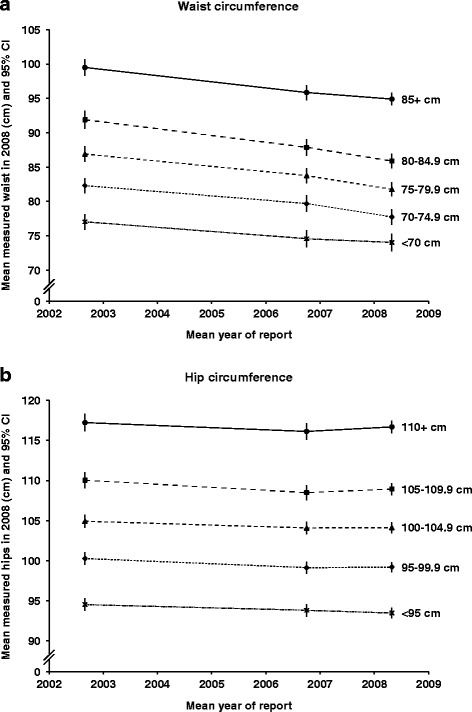
Mean measured values in 2008 by categories of self-reported values on three different occasions (2002, 2006, 2008) (**a**) waist circumference, (**b**) hip circumference

We also examined how differences between reported and measured values might affect estimates of associations of disease outcomes with categorical anthropometric variables during the 9-year period of this study (Figs. 1 and 2). Each graph presents the mean measured value from the examination in 2008, within each of five categories of self-reported values from previous questionnaires. Numerical values corresponding to these graphs are shown in the Web Table 3. For the four variables shown (weight, BMI, waist and hip circumference), lines are approximately parallel, demonstrating that there was little or no change over time in differences between mean measured values on each of the five categories. This reflects the lack of trend of regression dilution ratios over time, shown in Table 3. Height was only reported on two questionnaires, and is not shown in the figures; however, mean measured values within the five categories for height also show little change over time (Web Table 3).

For weight, BMI and hip circumference, the graphs also illustrate that categories of self-reports from each of the questionnaires are representative of similar mean values at the measurement, without strong trends by elapsed time between self-report and measurement (lines are close to horizontal for each category). The graph for waist circumference shows that categories of waist circumference based on first report (in 2002) are representative of a higher mean measured waist circumference at the measurement than are categories based on later reports, which is consistent with the more substantial changes in mean differences observed over time for waist circumference than for the other variables (shown in Table 3). Even so, the differences between the categories do not vary greatly with elapsed time between the questionnaire and the measurement; again, this is consistent with the lack of trend in regression dilution ratios.

## Discussion

### Key findings

In this large study of the validity of self-reported anthropometry over time, we found good evidence to support the use of such variables in large-scale prospective epidemiological studies with long-term follow-up. Weight, height, derived BMI, and waist and hip circumferences were well-reported on self-administered questionnaires at recruitment and during study follow-up. For all variables, reported values were lower on average than measured values (i.e. under-reported), except for height, for which reported values were higher on average (over-reported). Differences between self-reported and measured values were largest for the earliest reports of each variable, and became smaller the shorter the elapsed time between self-report and measurement. This is likely to be at least partly a consequence of real changes over time in these variables, since as women age their body fatness tends to increase [17–20], while their height tends to decrease [19, 20].

Differences between reported and measured anthropometric variables (due either to reporting errors or changes over time in actual characteristics) can bias estimates of linear associations with disease outcomes by regression dilution [16]. The magnitude of this regression dilution bias was not large for any variable, but was greatest for waist circumference: an approximately 20 % attenuation of regression coefficients would be expected in analyses of waist circumference (for example, a 20 % reduction in log relative risks would mean that a relative risk of 1.50 would be expected to be attenuated to 1.38). For all variables, regression dilution bias was stable over the 6–9 year period of this study. Overall, each of the self-reported anthropometric variables that we examined was informative about actual body size, and would therefore be suitable for use in epidemiological analyses. If appropriate, corrections for regression dilution can be applied to improve estimates of disease-exposure associations [15, 16].

When associations are non-linear, reporting errors may still affect estimates even when there is little regression dilution bias. For example, in assessing the lowest risk level of exposure in a U- or J-shaped relationship, if the exposure tends to be under-reported, as for weight, derived BMI, and waist and hip circumferences, then the lowest risk level will also tend to be underestimated. If the exposure tends to be over-reported, as for height, then the lowest risk level will tend to be overestimated. The mean differences between reported and measured values (Table 3) and the trends over time in mean measured values within categories of self-reports (Figs. 1 and 2) summarise these effects. With the exception of waist circumference, for which the mean difference was large and changed substantially over 6 years, the mean differences for the variables considered here were not large (relative to the standard deviation) and changes over time were also small in magnitude. For most anthropometric variables, the effects of under- or over-reporting on non-linear associations would therefore be modest.

Loss of power is another important consequence of differences between reported and measured anthropometric variables. Loss of power can be expressed as an effective sample size for studies based on self-reported anthropometric exposures, relative to those based on measured anthropometry, which can be estimated as the square of the correlation coefficient between self-reported and measured values [14, 15]. In this study, analyses of weight, height or BMI based on reported values would lose power equivalent to a reduction in sample size of 5 to 30 %, depending on the period of follow-up. For example, a cohort study with 1000 actual cases of the outcome of interest would have an effective sample size of between 700 and 950 cases, if reported weight or height or derived BMI are the exposures. Self-reported waist and hip circumference are less closely correlated with measured values. The effective reduction in sample size would be about 40 % for hip circumference and 50 % for waist circumference, so in the cohort study example above, the effective sample size would be only 500–600 cases. To avoid overestimating study power in analyses based on self-reported variables, power calculations should be based on distributions of self-reported data, with hypothesised effect sizes attenuated to account for realistic regression dilution bias.

### Previous studies

Other studies of self-reported anthropometric variables have generally found good agreement between values reported on a single occasion and contemporaneous measured data. Most other studies found a slight over-reporting of height and slight to moderate under-reporting of weight, BMI, waist and hip circumference, and strong to very strong correlations between self-reported and measured values, consistent with our findings [1–9]. We have also previously reported the validity of these anthropometric variables at a single time point for 541 Million Women Study participants who were matched to a UK birth cohort study [10]. The mean differences, correlation coefficients and regression dilution ratios that we report here are all consistent with those found in our earlier study.

We were unable to identify any other study that has assessed the validity over time of repeated self-reported values. The Emerging Risk Factors Collaboration did report regression dilution ratios for repeated assessments of height, BMI, waist and hip circumference, but most of the contributing cohorts obtained anthropometric information by measurement, and those cohorts with self-reported values were not examined separately [11, 12]. The regression dilution ratios that they found for these predominantly measured variables were generally close to 1 (0.86 to 0.96) and stable over an average of six years of follow-up.

### Strengths and limitations

Our study included matched self-reported and measured anthropometry on a large sample of women from a national UK cohort. Self-reports were available for this sample from four questionnaires over a 9 year follow-up period. Average completeness of these data across the questionnaires was high: reported weight and height were 97 and 99 % complete, respectively, and reported waist and hip circumference were each 75 % complete among women who were asked to report these variables. Measured values were available for >99 % of these women for all variables.

Women were selected at random, and those who agreed to participate were similar to the full study cohort. Women were chosen from areas in England and Scotland to ensure broad geographical coverage. As is common in the large-scale epidemiological studies to which our findings are relevant, regression analyses were adjusted for age and region of recruitment. A potential limitation of this study was that anthropometric measurements were taken at only one point in time, so we could not directly assess changes in measured body size over time. However, our findings were consistent with other data which suggest that as middle-aged women get older, weight, BMI and waist circumference tend to increase [17–20], and height tends to decrease [19, 20]. Our findings may not be applicable to men or to women in other age groups.

## Conclusion

Self-reported anthropometric variables differ from corresponding measurements due to random and systematic reporting errors, and to changes over time in the actual characteristics of participants. These differences may bias estimates of disease-exposure associations, although these biases appear to be small and stable over time for most variables. Reporting errors may also reduce effective study power. Overall, however, we found that self-reported weight, height, derived BMI, and waist and hip circumferences are suitable for use in epidemiological analyses with long-term follow-up.

## Additional file

Additional file 1:
**Validity over time of self-reported anthropometric variables during follow-up of a large cohort of UK women.** (DOCX 38 kb)

## Abbreviations

BMI: Body mass index
NHS: National health service

## References

[1] Rowland ML (1990). Self-reported weight and height. Am J Clin Nutr.

[2] Stevens J, Keil JE, Waid LR, Gazes PC (1990). Accuracy of current, 4-year, and 28-year self-reported body weight in an elderly population. Am J Epidemiol.

[3] Casey VA, Dwyer JT, Berkey CS, Coleman KA, Gardner J, Valadian I (1991). Long-term memory of body weight and past weight satisfaction: a longitudinal follow-up study. Am J Clin Nutr.

[4] Spencer EA, Appleby PN, Davey GK, Key TJ (2002). Validity of self-reported height and weight in 4808 EPIC-Oxford participants. Public Health Nutr.

[5] Spencer EA, Roddam AW, Key TJ (2004). Accuracy of self-reported waist and hip measurements in 4492 EPIC-Oxford participants. Public Health Nutr.

[6] Han TS, Lean MEJ (1998). Self-reported waist circumference compared with the ‘Waist Watcher’ tape-measure to identify individuals at increased health risk through intra-abdominal fat accumulation. Br J Nutr.

[7] Engstrom JL, Paterson SA, Doherty A, Trabulsi M, Speer KL (2003). Accuracy of self-reported height and weight in women: An integrative review of the literature. J Midwif Wom Heal.

[8] Gorber SC, Tremblay M, Moher D, Gorber B (2007). A comparison of direct vs. self-report measures for assessing height, weight and body mass index: a systematic review. Obes Rev.

[9] Stommel M, Schoenborn C (2009). Accuracy and usefulness of BMI measures based on self-reported weight and height: findings from the NHANES & NHIS 2001–2006. BMC Public Health.

[10] Cairns BJ, Liu B, Clennell S, Cooper R, Reeves GK, Beral V (2011). Lifetime body size and reproductive factors: comparisons of data recorded prospectively with self reports in middle age. BMC Med Res Methodol.

[11] The Emerging Risk Factors Collaboration (2011). Separate and combined associations of body-mass index and abdominal adiposity with cardiovascular disease: collaborative analysis of 58 prospective studies. Lancet.

[12] The Emerging Risk Factors Collaboration (2012). Adult height and the risk of cause-specific death and vascular morbidity in 1 million people: individual participant meta-analysis. Int J Epidemiol.

[13] Million Women Study Collaborators (2003). Breast cancer and hormone-replacement therapy in the Million Women Study. Lancet.

[14] McKeown-Eyessen GE, Tibshirani R (1994). Implications of measurement error in exposure for the sample size of case–control studies. Am J Epidemiol.

[15] Kaaks R, Riboli E, van Staveren W (1995). Calibration of dietary intake measurements in prospective cohort studies. Am J Epidemiol.

[16] Rosner B, Willett WC, Spiegelman D (1989). Correction of logistic regression relative risk estimates and confidence intervals for systematic within person measurement error. Stat Med.

[17] Ekelund U, Besson H, Luan J, May SM, Sharp SJ, Brage S (2011). Physical activity and gain in abdominal adiposity and body weight: prospective cohort study in 288,498 men and women. Am J Clin Nutr.

[18] Sternfeld B, Wang H, Quesenberry CP, Abrams S, Everson-Rose SA, Greendale GA (2004). Physical activity and changes in weight and waist circumference in midlife women: Findings from the Study of Women’s Health Across the Nation. Am J Epidemiol.

[19] Moayyeri A, Luben RN, Bingham SA, Welch AA, Wareham NJ, Khaw K-T (2008). Measured height loss predicts fractures in middle-aged and older men and women: the EPIC-Norfolk prospective population study. J Bone Miner Res.

[20] Droyvold WB, Nilsen TIL, Kruger O, Holmen TL, Krokstad S, Midthjell K (2006). Change in height, weight and body mass index: Longitudinal data from the HUNT Study in Norway. Int J Obesity.

