# Supplementary material for: Validity over time of self-reported anthropometric variables during follow-up of a large cohort of UK women
Source: BMC Med Res Methodol. 2015 Oct 8;15:81. doi: 10.1186/s12874-015-0075-1 (PMC4599695; doi:10.1186/s12874-015-0075-1)
Supplement: Additional file 1: — Validity over time of self-reported anthropometric variables during follow-up of a large cohort of UK women. (DOCX 38 kb) [file 12874_2015_75_MOESM1_ESM.docx]

# **ADDITIONAL MATERIALS**

Validity over time of self-reported anthropometric variables during follow-up of a large cohort of UK women

Table of contents

**Methods supplement** …………………………………….……………………………………….……………….……….. ***Pages 2-3***

**Web Table 1**. Completeness of self-reported and measured anthropometric data of 3999 women in the study sample. ……………………….…………..……………………….…….….…………………….…………………….. ***Page 4***

**Web Table 2**. Self-reported anthropometric characteristics in the entire Million Women Study cohort. …………………………………………………………………………………………………………………………….……… ***Page 5***

**Web Table 3**. Mean measured values in 2008 by categories of self-reported values on up to four occasions (1999, 2002, 2006, 2008). …...……………………………………….…………….………...…………..…. ***Page 6***

**METHODS SUPPLEMENT**

Inclusion Criteria

The 2006 (third) study questionnaire was used to identify women for this study, which was part of a programme of clinical investigations (see www.millionwomenstudy.org/dss_summary). We included women who had been randomly pre-selected from the main cohort of the Million Women Study and had returned a completed 2006 study questionnaire. From these women, we included those who had not reported a diagnosis of breast cancer or vascular disease (ischaemic heart disease, cerebrovascular disease or venous thromboembolism) in the previous 5 years, and, were registered at the time with a general practitioner in Scotland or in selected NHS Comprehensive Local Research Network areas of England, chosen with the aim of ensuring broad geographical coverage. The included areas in England were: Northumberland, Tyne and Wear; County Durham and Tees Valley; North East Yorkshire & Northern Lincolnshire; Essex and Hertfordshire; Thames Valley; Surrey and Sussex; Kent and Medway.

Data Collection

*Questionnaire*

Between 2006 and 2008, 14762 women who met the above criteria were sent an additional questionnaire. All were asked questions about their current weight and height. Women resident in Scotland were contacted first, after which questions on waist and hip circumference were added to the study questionnaire; only women resident in England at the time of the study were asked about their current waist and hip circumferences. A completed questionnaire was returned by 9278 (63%) women and of those, 5975 (64% of 9278) indicated that they were willing to attend a general practice appointment for clinical investigations.

*Measurement*

We wrote to each woman’s registered general practitioner asking them to inform us within a month if they preferred not to have the woman approach the surgery for a clinical examination. Approximately 1% (70/5975) of women were registered with a general practitioner who declined to participate, and these women were therefore excluded from the study. We wrote to the remaining women asking them to make an appointment at their general practice with either a practice nurse or general practitioner to have their anthropometric measurements taken. A study form with brief instructions and a section to record clinical measurements was included for the practice staff to complete and the woman to return in a prepaid envelope (for weight and height: ‘patient should be wearing light indoor clothes with emptied pockets and without shoes’). Women resident in England were sent a tape measure to take to the appointment to ensure that the practice staff had standardized equipment to perform waist and hip measurements. For these women, additional instructions for waist and hip measurement were included on the study form (‘measure the waist at the level of the navel/ umbilicus; measure hip circumference at the widest point of the buttocks’). Between December 2006 and February 2010, 3999 women attended a general practice appointment for the study’s clinical investigations, returned a completed study form with recorded measurements, and had usable reported values. The majority (64%) were resident in England.

Data processing

Each woman’s 2008 questionnaire was matched with the corresponding clinical form from the general practice appointment. Anthropometric data from each were entered independently by two data entry clerks. Discrepancies and extreme values for each variable were checked against the questionnaire or study form and corrected or excluded as necessary by the lead researcher (FLW). Self-reported values for anthropometric variables from the 1999, 2002 and 2006 study questionnaires were obtained from the Million Women Study database.

**Web Table 1. Completeness of self-reported and measured anthropometric data of 3999 women in the study sample**

|  | **Mean year of report** | | | | **Mean year of measurement** |
| --- | --- | --- | --- | --- | --- |
|  | **1999^a^** | **2002**^a,b^ | **2006^a^** | **2008** | **2008** |
| Weight, % (n) | 98 (3908) | 96 (3541) | 97 (3885) | 98 (3935) | 99.5 (3980) |
| Height^c^ % (n) | 99 (3970) | NA | NA | 99 (3961) | 99.6 (3983) |
| BMI^c^, % (n) | 97 (3887) | 95 (3516) | 97 (3857) | 98 (3909) | 99.4 (3974) |
| Waist, % (n) | NA | 74 (2715) | 67 (2695) | 84 (2156)^d^ | 99.9 (2573)^d^ |
| Hips, % (n) | NA | 74 (2732) | 67 (2691) | 84 (2174)^d^ | 99.5 (2564)^d^ |

Abbreviation: BMI, body mass index; NA, not asked

a) 1999 is 1^st^ (recruitment) questionnaire, 2002 is 2^nd^ questionnaire, and 2006 is 3^rd^ questionnaire

b) 3691 of 3999 women responded to this questionnaire

c) BMI values for 2002 and 2006 were calculated using height reported in 1999.

d) Only women resident in England (N=2576) were asked to report waist & hip measurements on the 2008 questionnaire & to have these measured.

**Web Table 2. Self-reported anthropometric characteristics in the entire Million Women Study cohort**

|  | **Mean year of report** | | |
| --- | --- | --- | --- |
|  | **1998** | **2001** | **2006** |
| Women, N | 1,364,367 | 866,536 | 683,244 |
| Age at time of report (SD) | 56.2 (4.9) | 59.6 (5.0) | 63.9 (4.8) |
| **Anthropometry:** |  |  |  |
| Weight, kg, mean (SD) | 68.8 (12.7) | 68.8 (12.5) | 69.7 (13.0) |
| Height, cm, mean (SD) | 162.0 (6.7) | NA | NA |
| BMI, kg/m^2^, mean (SD)^a^ | 26.2 (4.7) | 26.2 (4.6) | 26.5 (4.8) |
| Waist, cm, mean (SD) | NA | 76.6 (9.5) | 79.3 (10.2) |
| Hips, cm, mean (SD) | NA | 100.4 (7.6) | 100.9 (8.0) |

Abbreviations: BMI, body mass index; NA, not asked; SD, standard deviation.

a) BMI values for 2001 & 2006 were calculated using height reported in 1999.

**Web Table 3. Mean measured values in 2008 by categories of self-reported values on up to four different occasions (1999, 2002, 2006, 2008)^a^**

|  | **Mean measured values (se)^b^ in 2008 for different years of self-report** | | | |
| --- | --- | --- | --- | --- |
| **Reported characteristic** | 1999 | 2002 | 2006 | 2008 |
| **Weight** (kg), **N** | **3893** | **3530** | **3870** | **3921** |
| 85+ | 94.8 (0.5) | 94.5 (0.4) | 94.2 (0.4) | 95.3 (0.3) |
| 75.0-84.9 | 82.1 (0.4) | 81.3 (0.4) | 80.2 (0.3) | 80.6 (0.3) |
| 65.0-74.9 | 72.4 (0.3) | 71.6 (0.3) | 71.0 (0.3) | 71.2 (0.2) |
| 55.0-64.9 | 63.0 (0.3) | 62.0 (0.3) | 61.6 (0.3) | 61.7 (0.2) |
| <55.0 | 53.8 (0.4) | 53.1 (0.4) | 52.1 (0.4) | 52.2 (0.3) |
| ***Range*** | ***41.0*** | ***41.4*** | ***42.1*** | ***43.1*** |
| **Height** (cm), **N** | **3951** |  |  | **3943** |
| 170+ | 170.4 (0.2) |  |  | 170.8 (0.2) |
| 165-169.9 | 165.2 (0.2) |  |  | 165.6 (0.1) |
| 160-164.9 | 160.7 (0.2) |  |  | 161.2 (0.1) |
| 155-159.9 | 156.8 (0.2) |  |  | 157.3 (0.2) |
| <155 | 153.2 (0.2) |  |  | 152.9 (0.2) |
| ***Range*** | ***17.2*** |  |  | ***17.9*** |
| **BMI** (kg/m^2^), **N** | **3863** | **3496** | **3834** | **3887** |
| 30+ | 34.5 (0.2) | 34.3 (0.2) | 34.0 (0.1) | 34.2 (0.1) |
| 27.5-29.9 | 30.3 (0.2) | 29.8 (0.2) | 29.7 (0.1) | 29.5 (0.1) |
| 25-27.4 | 27.7 (0.1) | 27.3 (0.1) | 26.9 (0.1) | 27.1 (0.1) |
| 22.5-24.9 | 25.2 (0.1) | 24.8 (0.1) | 24.6 (0.1) | 24.5 (0.1) |
| <22.5 | 22.2 (0.1) | 22.0 (0.1) | 21.7 (0.1) | 21.5 (0.1) |
| ***Range*** | ***12.3*** | ***12.3*** | ***12.3*** | ***12.7*** |
| **Waist** (cm), **N** |  | **1771** | **1717** | **2153** |
| 85.0+ |  | 99.5 (0.6) | 95.9 (0.6) | 94.9 (0.4) |
| 80.0-84.9 |  | 91.9 (0.7) | 87.9 (0.6) | 85.9 (0.5) |
| 75.0-79.9 |  | 86.9 (0.6) | 83.8 (0.6) | 81.8 (0.5) |
| 70.0-74.9 |  | 82.3 (0.6) | 79.7 (0.6) | 77.7 (0.6) |
| <70.0 |  | 77.0 (0.6) | 74.6 (0.6) | 74.0 (0.7) |
| ***Range*** |  | ***22.5*** | ***21.3*** | ***20.9*** |
| **Hips** (cm), **N** |  | **1773** | **1719** | **2165** |
| 110.0+ |  | 117.2 (0.6) | 116.1 (0.5) | 116.7 (0.4) |
| 105.0-109.9 |  | 110.0 (0.5) | 108.5 (0.5) | 108.9 (0.4) |
| 100.0-104.9 |  | 104.9 (0.4) | 104.1 (0.4) | 104.1 (0.4) |
| 95.0-99.9 |  | 100.3 (0.4) | 99.1 (0.4) | 99.2 (0.3) |
| <95 .0 |  | 94.5 (0.4) | 93.8 (0.4) | 93.5 (0.3) |
| ***Range*** |  | ***22.7*** | ***22.3*** | ***23.2*** |

Abbreviation: se, standard error

a) Data illustrated in Figures 1 and 2.

b) Adjusted for age at measurement and recruitment region.
